# Medicinal Plants: Advances in Phytochemistry and Ethnobotany

**DOI:** 10.3390/plants12081682

**Published:** 2023-04-17

**Authors:** Dâmaris Silveira, Fabio Boylan

**Affiliations:** 1 Department of Pharmacy, Faculty of Health Sciences, University of Brasilia, Brasilia 70910-900, Brazil; 2 School of Pharmacy and Pharmaceutical Sciences and Trinity Biomedical Sciences Institute, Trinity College Dublin, D02 PN40 Dublin, Ireland

Prance once defined Ethnobotany as an interdisciplinary Science combining Anthropology and Botany [1]. However, it can also involve Phytochemistry, Pharmacology, Nutrition, and other uses of plants by a traditional community. Furthermore, Ethnobotanical studies often consider ecological aspects of the traditional use of such plants, either because of their importance for the community environment or the non-rational exploitation of some species. Thus, Ethnobotany can bring together the know-how of a local community and scientific knowledge, contributing to achieving biocultural conservation [2]. Moreover, it can contribute to carrying plants from local markets to a global trade situation, valuing and preserving traditional knowledge.

In 2022, the global herbal medicines market was worth USD 170 billion, and the expectation is that this market has the potential to reach USD 600 billion by 2033, with a compound annual growth rate (CAGR) of 15% from 2023 to 2033 [3]. Considering dry herbs, such as oregano, rosemary, sage, savoury, mint, thyme, and bay leaves in the form of the whole plant or powdered material, the global market grew from USD 5.8 billion in 2022 to USD 6.17 billion in 2023 (a CAGR of 6.3%), and there is an expectation it will reach USD 7.93 billion in 2027 [4]. Considering more elaborate forms, such as capsules, tablets, and extracts, the global market forecast is USD 117 billion by 2029, with a CAGR of 7.3% [5].

This Special Issue of *Plants*, dedicated to Ethnobotany and Phytochemistry, received 27 manuscript submissions from almost all world regions. From those, 13 papers were of a high quality and were published. They comprise a wide range of Ethnobotany or Phytochemical aspects, mostly involving native species.

Monari et al. (2022) analysed published Italian studies involving Ethnobotany and medicinal plants and collected data from 1117 species from 75 papers. The information helps to develop and preserve knowledge of those plants [6]. Through an ethnobotanical survey, Odebunmi et al. (2022) registered species from 29 families used by Nigerian people to treat COVID-19 and related symptoms (flu and cough). The most cited plants by the 56 participants of the study were *Zingiber officinale* Roscoe and *Citrus limon* (L.) Osbeck [7]. Berlowitz et al. (2023) described the medicinal use of *Nicotiana rustica *L. to treat a woman with several mental disorders in the Peruvian Amazon [8].

The family Amaryllidaceae was the subject of two studies. In the first paper, Tallini et al. (2022) described the chemical profile of three Peruvian species from the genus *Rauhia* [9]. The authors identified 30 different alkaloids by GC-MS in the extracts of *Rauhia staminosa *Ravenna, *R. decora *Ravenna, and *R. multiflora *Ravenna. *Rauhia multiflora* presented the highest acetylcholinesterase inhibition, followed by *R. staminosa* and *R. decora. *In the second paper, Gomes-Copeland et al. (2022) showed the activity of *Hippeastrum stapfianum* (Kraenzl.) R.S.Oliveira & Dutilh, a Brazilian species, on acetylcholinesterase inhibition and interacting with the nuclear receptors PPAR-α and PPAR-γ [10].

Carneiro et al. (2022) described an extract of *Morus nigra* L. presenting agonism on both PPAR-α and PPAR-γ and its capacity to reduce the production of ROS, NO, and TNF-α on RAW 264.7 cells [11].

The isolation of natural compounds presenting pharmacological activity plays an important role in the valorisation of plants traditionally used as food or medicine. The cytotoxic activity of tylophorinidine, a phenanthroindolizidine alkaloid isolated from *Tylophora indica* (Burm.f.) Merr, an Indian native species, was described by Mostafa et al. (2022). The compound presented IC_50_ values of 6.45, 4.77, and 20.08 μM in MCF-7, HepG2, and HCT-116 cell lines, respectively [12]. The in vitro cytotoxic and anti-migratory effects of extracts from *Marantodes pumilum* Blume Kuntze, a Malaysian plant, as well as of an isolated compound from the chloroform fraction, 5-henicosene-1-yl-resorcinol, on prostate cancer cells (PC3) was shown by Hanafi et al. (2023). The authors found that the mechanism of action involves apoptosis, inhibition of both migration and invasion, and inhibition of angiogenesis [13].

Souza et al. (2023) showed the diuretic action of hesperidin, a flavanone glycoside from *Citrus* fruits, in hypertensive rats. The authors suggested that the activity is associated with the cholinergic pathway [14].

Essential oil has played a vital role in Ethnobotany since ancient times. Alsharif et al. (2022) characterised the volatiles in the leaves of *Capparis cartilaginea* Decne from Saudi Arabia. The GC-MS analysis led to the identification of isopropyl isothiocyanate, 2-methylbutanenitrile, 2-butyl isothiocyanate, isobutyronitrile, and 3-methyl-butane nitrile [15]. Neves et al. (2022) evaluated the essential oil from the Brazilian *Campomanesia lineatifolia* Ruiz & Pav. on four *Helicobacter pylori* strains. The essential oil inhibited the growth of all strains, with an MIC = 6 L/mL [16]. Additionally, the essential oil from *Pulicaria dysenterica* (L.) Bernh., collected in Serbia, was tested on acute toxicity, antimicrobial and antispasmodic activity, acetylcholinesterase inhibition, and cytotoxic properties by Radulovic et al. (2022). The authors identified a new natural compound (3-methoxycuminyl 2-methylbutanoate) and another rare one (3-methoxycuminyl 3-methylbutanoate) [17].

Finally, Sarapan et al. (2023) discussed some botanical aspects of *Disporopsis longifolia* Craib, a traditional Asian medicinal plant. The findings of this research are useful for the quality control of this plant drug [18].

Therefore, if well conducted, Ethnobotany research and the interaction between academics and traditional communities can help to preserve biodiversity, improve the local economy, and rescue and protect traditional knowledge.

## References

[1] Prance G.T. (1991). What is ethnobotany today?. J. Ethnopharmacol..

[2] Pei S., Alan H., Wang Y. (2020). Vital roles for ethnobotany in conservation and sustainable development. Plant Divers..

[3] Newsmantraa Herbal Medicines Market: The Global Market for Herbal Medicines Is Expected to Reach $600 Billion by the End of 2033. https://www.reportlinker.com/p06031785/?utm_source=GNW.

[4] ReporterLinker Dried Herbs Global Market Report 2023. https://www.globenewswire.com/news-release/2023/03/07/2622091/0/en/Dried-Herbs-Global-Market-Report-2023.html.

[5] Jangir M., Global Herbal Supplements Market Is Anticipated to Grow at a CAGR of 7.3% during 2022-29 The Market Would Reach USD 117.8 Bn by 2029. https://www.cbs42.com/business/press-releases/ein-presswire/619479991/herbal-supplements-market-worth-117-8-billion-by-2029-at-a-growth-rate-of-7-3/.

[6] Monari S., Ferri M., Salinitro M., Tassoni A. (2022). Ethnobotanical Review and Dataset Compiling on Wild and Cultivated Plants Traditionally Used as Medicinal Remedies in Italy. Plants.

[7] Odebunmi C.A., Adetunji T.L., Adetunji A.E., Olatunde A., Oluwole O.E., Adewale I.A., Ejiwumi A.O., Iheme C.E., Aremu T.O. (2022). Ethnobotanical Survey of Medicinal Plants Used in the Treatment of COVID-19 and Related Respiratory Infections in Ogbomosho South and North Local Government Areas, Oyo State, Nigeria. Plants.

[8] Berlowitz I., García Torres E., Maake C., Wolf U., Martin-Soelch C. (2023). Indigenous-Amazonian Traditional Medicine’s Usage of the Tobacco Plant: A Transdisciplinary Ethnopsychological Mixed-Methods Case Study. Plants.

[9] Tallini L.R., Osorio E.H., Berkov S., Torras-Claveria L., Rodríguez-Escobar M.L., Viladomat F., Meerow A.W., Bastida J. (2022). Chemical Survey of Three Species of the Genus Rauhia Traub (Amaryllidaceae). Plants.

[10] Gomes-Copeland K.K.P., Meireles C.G., Gomes J.V.D., Torres A.G., Sinoti S.B.P., Fonseca-Bazzo Y.M., Magalhães P.d.O., Fagg C.W., Simeoni L.A., Silveira D. (2022). Hippeastrum stapfianum (Kraenzl.) R.S.Oliveira & Dutilh (Amaryllidaceae) Ethanol Extract Activity on Acetylcholinesterase and PPAR-alpha/gamma Receptors. Plants.

[11] Carneiro A.A., Sinoti S.B.P., Freitas M.M., Simeoni L.A., Fagg C.W., Magalhães P.O., Silveira D., Fonseca-Bazzo Y.M. (2022). Hydroethanolic Extract of Morus nigra L. Leaves: A Dual PPAR-α/γ Agonist with Anti-Inflammatory Properties in Lipopolysaccharide-Stimulated RAW 264.7. Plants.

[12] Mostafa E.M., Mohammed H.A., Musa A., Abdelgawad M.A., Al-Sanea M.M., Almahmoud S.A., Ghoneim M.M., Gomaa H.A.M., Rahman F.E.-Z.S.A., Shalaby K. (2022). In Vitro Anti-Proliferative, and Kinase Inhibitory Activity of Phenanthroindolizidine Alkaloids Isolated from Tylophora indica. Plants.

[13] Hanafi M.M.M., Yaakob H., Gibbons S., Prieto J.M. (2023). In Vitro Pro-Apoptotic and Anti-Migratory Effects of Marantodes pumilum (syn. Labisia pumila) Extracts on Human Prostate Cancer Cell Lines: Bioguided Isolation of 5-Henicosene-1-yl-resorcinol. Plants.

[14] Souza P., Silva R.d.C.V., Mariano L.N.B., Dick S.L., Ventura G.C., Cechinel-Filho V. (2023). Diuretic and Natriuretic Effects of Hesperidin, a Flavanone Glycoside, in Female and Male Hypertensive Rats. Plants.

[15] Alsharif B., Babington G.A., Radulović N., Boylan F. (2022). Volatiles of Capparis cartilaginea Decne. from Saudi Arabia. Plants.

[16] Neves N.C.V., Mello M.P., Smith S.M., Boylan F., Caliari M.V., Castilho R.O. (2022). Chemical Composition and In Vitro Anti-*Helicobacter pylori* Activity of *Campomanesia lineatifolia* Ruiz & Pavón (Myrtaceae) Essential Oil. Plants.

[17] Radulović N.S., Mladenović M.Z., Vukićević D.R., Stojanović N.M., Randjelović P.J., Stojanović-Radić Z.Z., Boylan F. (2022). *Pulicaria dysenterica* (L.) Bernh.—Rightfully Earned Name? Identification and Biological Activity of New 3-Methoxycuminyl Esters from *P. dysenterica* Essential Oil. Plants.

[18] Sarapan A., Hodkinson T.R., Suwanphakdee C. (2023). Assessment of Morphological, Anatomical and Palynological Variation in the Medicinal Plant Disporopsis longifolia Craib (Asparagaceae) for Botanical Quality Control. Plants.

